# Fibronectin induces a transition from amoeboid to a fan morphology and modifies migration in *Entamoeba histolytica*

**DOI:** 10.1371/journal.ppat.1012392

**Published:** 2024-07-25

**Authors:** Maria Manich, Pascal Bochet, Aleix Boquet-Pujadas, Thierry Rose, Gertjan Laenen, Nancy Guillén, Jean-Christophe Olivo-Marin, Elisabeth Labruyère

**Affiliations:** 1 Institut Pasteur, Université de Paris Cité, Biological Image Analysis Unit, Paris, France; 2 Centre National de la Recherche Scientifique, CNRS-UMR3691, Paris, France; 3 École Polytechnique Fédérale de Lausanne, Biomedical Imaging Group, Lausanne, Switzerland; 4 Institut Pasteur, Diagnostic Test Innovation and Development Core Facility Unit, Paris, France; 5 Centre National de la Recherche Scientifique, CNRS-ERL9195, Paris, France; University of California Davis, UNITED STATES OF AMERICA

## Abstract

Cell migration modes can vary, depending on a number of environmental and intracellular factors. The high motility of the pathogenic amoeba *Entamoeba histolytica* is a decisive factor in its ability to cross the human colonic barrier. We used quantitative live imaging techniques to study the migration of this parasite on fibronectin, a key tissue component. *Entamoeba histolytica* amoebae on fibronectin contain abundant podosome-like structures. By using a laminar flow chamber, we determined that the adhesion forces generated on fibronectin were twice those on non-coated glass. When migrating on fibronectin, elongated amoeboid cells converted into fan-shaped cells characterized by the presence of a dorsal column of F-actin and a broad cytoplasmic extension at the front. The fan shape depended on the Arp2/3 complex, and the amoebae moved laterally and more slowly. Intracellular measurements of physical variables related to fluid dynamics revealed that cytoplasmic pressure gradients were weaker within fan-shaped cells; hence, actomyosin motors might be less involved in driving the cell body forward. We also found that the *Rho-associated coiled-coil containing protein kinase* regulated podosome dynamics. We conclude that *E*. *histolytica* spontaneously changes its migration mode as a function of the substrate composition. This adaptive ability might favour *E*. *histolytica*’s invasion of human colonic tissue. By combining microfluidic experiments, mechanical modelling, and image analysis, our work also introduces a computational pipeline for the study of cell migration.

## Author summary

Even though we know increasingly well how cells move in tissues, the question of whether parasites modify their migration as a function of the host tissue environment requires further investigation. Cell motility is triggered by environmental stimuli, and the migration mode depends on adhesion, activated enzymatic pathways, and changes in the actin cytoskeleton dynamics and thus cell morphology. The protozoan intestinal parasitic *Entamoeba histolytica* is the causative agent of amoebiasis (a major food- and water-borne disease) in humans. During invasion of the colon, *E*. *histolytica* degrades the mucus layer, adheres to and removes the epithelium, progresses along the fibronectin-rich basement membrane towards the crypts of Lieberkühn, and penetrates the mucosa. This invasive process raises the question of how the parasite moves in a varying intestinal environment. *E*. *histolytica* is known to display an amoeboid migration mode and binds fibronectin through podosome adhesion structures. We hypothesized that fibronectin triggers signalling pathways that influence the trophozoite’s migration. We found that *E*. *histolytica* can migrate with a non-amoeboid mode on fibronectin-coated glass. Our finding suggests that for tissue invasion, *E*. *histolytica*’s migration mode is modulated to suit the environment.

## Introduction

To navigate through tissues, mammalian cells sense the substrate using cell adhesion complexes. Extracellular matrix (ECM) receptors allow motile cells to respond to environmental changes. Motile cells change their morphology in three phases: (i) cell polarization, with membrane protrusions at the leading edge, (ii) interaction with the environment via adhesive structures that may contain signalling receptors, and (iii) translocation of the centre of mass by retracting the rear end [1,2]. The molecular repertoire responsible for cell polarization induces different shapes and movements which, in turn, make it possible to classify the mode of cell migration as mesenchymal or amoeboid [3].

The forces produced by the activity of the actin-rich cytoskeleton are essential for mesenchymal motility, in which the growing, barbed end of microfilaments pushes on the cell cortex, and the Arp2/3 protein complex promotes the nucleation and growth of new actin filaments. Mesenchymal motile cells have an elongated shape and are recognizable by their broad migration front (lamellipodia) and spike-shaped structures (filopodia) at the leading edge. In contrast, the presence of a pseudopod at the front of the cell and actomyosin contractility at the rear are the hallmarks of amoeboid migration. In the latter, local membrane stresses and internal pressure gradients make the cell highly deformable [3].

Mesenchymal and amoeboid migration modes are not mutually exclusive, and transitions from mesenchymal to amoeboid migration are promoted by weak cell adhesion; this is correlated with an induction of myosin-II activity by the small-GTPase Rho and/*or the Rho-associated coiled-coil containing protein kinases* (ROCKs) [4]. Amoeboid migration encompasses a broad spectrum of motion because the cell’s shape can adapt rapidly in various environments [5]. For example, morphometric analysis has shown that leukocytes have an intrinsic shape deformation ability, depending on to the substrate’s physical properties. In particular, the leukocyte’s traveling speed is correlated with the speed of actin flow, the dynamics of which depend on the shear forces required to move the cell body forward and are induced by the substrate’s texture [6]. These data support the hypothesis whereby the forward migration of amoeboid cells depends on rapid adaptation to varying environments.

Like mammalian motile cells, single-celled protists (e.g. choanoflagellates and amoebozoans) are not limited to a single migration mode. For example, the choanoflagellate *Salpingoeca rosetta* (often studied as a model of premetazoan evolution) reversibly switches from a flagellate to an amoeboid upon physical confinement. This cellular transition involves the activation of myosin-II-based motility [7]. In the social amoeba *Dictyostelium discoideum*, the pseudopod extension-retraction cycle operates during three types of movement: walking, gliding, and swimming [8]. In addition to pseudopod-based amoeboid motility, *D*. *discoideum* mutants can move in a manner reminiscent of kidney-shaped or so-called fan-shaped cells [9,10].

In the present study, we investigated locomotion in the ancient amoeba parasite *Entamoeba histolytica–*the causative agent of amoebiasis. In humans, the infectious process leading to tissue invasion is strongly dependent on the parasite’s motility. To invade the human colon, the trophozoite (the vegetative form of this parasite) crosses various intestinal environments and structures, such as the mucus, epithelia, the ECM, and blood vessels [11,12]. After degradation of the mucus layer and the removal of the epithelial cells, the parasite migrates over the fibronectin (FN)-rich basement membrane towards the crypts of Lieberkühn and then penetrates into the collagen-I-fibre-rich ECM [13,14]. This amoeboid migration is characterized by changes in cell shape: an initial exploratory stage is characterized by pseudorandom blebbing [15] and is followed by the growth of a pseudopod at the front of the trophozoite [16] and a uropod at the rear of the cell. The uropod contains the actomyosin cytoskeleton and actin-binding proteins [17] required to retract the cell body and thus push the trophozoite forward [16,18]. The dynamics of the actomyosin cytoskeleton constitute a key element in the genesis of force for migration [19–21]. The measurement of intracellular biophysical variables has confirmed that (i) forces are generated at the rear of the cell and (ii) *E*. *histolytica*’s amoeboid movement is driven by pressure [18]. To date, *E*. *histolytica*’s migratory behaviour has been mainly studied on glass supports and in three-dimensional matrices of collagen I fibres [13]. As mentioned above, the parasite migrates over the FN-rich basement membrane in the intestine. *In vitro* studies have shown that when *E*. *histolytica* is loaded on an FN substrate, the parasite develops structures resembling the podosomes of mammalian cells [22–24]. It has been suggested that *E*. *histolytica* interacts with FN via a β1-integrin-like receptor [25,26]. A more detailed view of the molecular processes that regulate amoeba migration should improve our understanding of the infectious process. In the present study, we looked at whether an interaction with FN influences *E*. *histolytica*’s adhesion and migration. We mainly used live imaging and specific cytoskeleton inhibitors to study the impact of FN on the parasite’s behaviour. Our findings demonstrate that the FN-induced signalling pathway leads to a change in the parasite’s morphodynamics: the migration mode differs from an amoeboid movement, and the cells become fan-shaped. More generally, we highlighted *E*. *histolytica’s* ability to adapt its migration to suit the extracellular environment and developed a general computational pipeline for studying cell migration.

## Results

### The adhesion force of *Entamoeba histolytica* on FN is twice that on glass

**Fig 1:**
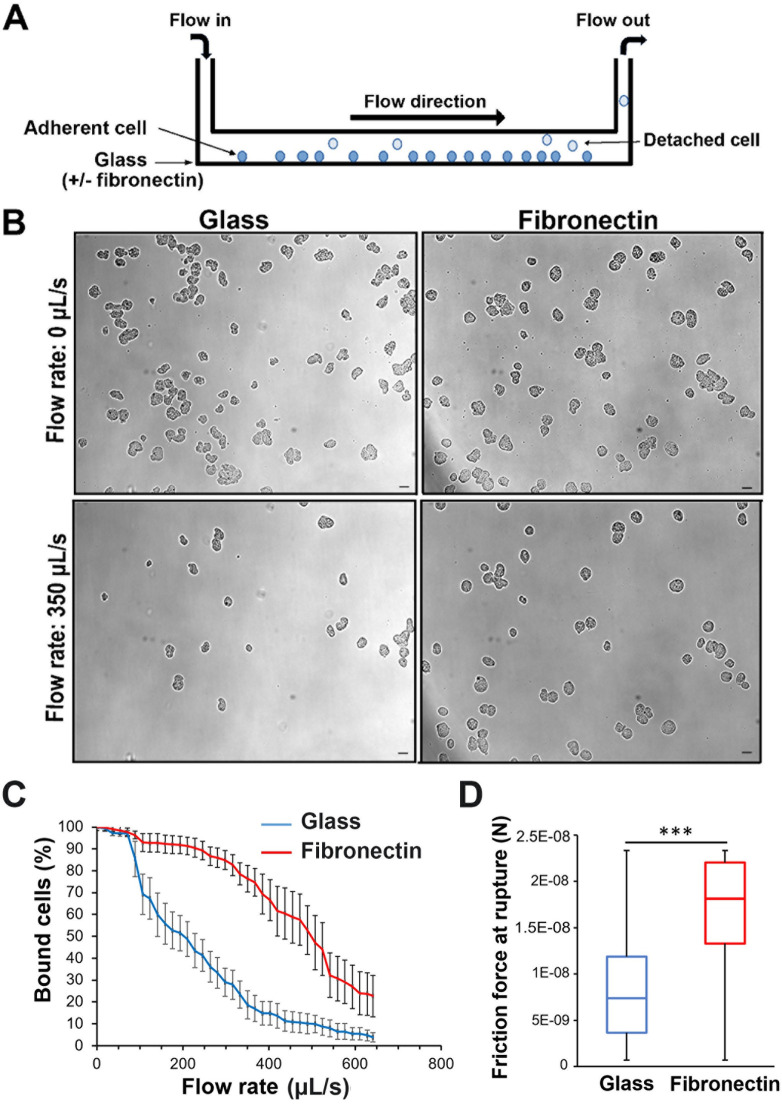
*E*. *histolytica* adhesion forces are stronger on FN than on glass. **A.** Schematic representation of a laminar flow chamber. **B.** Representative images of adherent amoeba on non-coated glass and on FN-coated glass in the absence of flow (T0) and at a flow rate of 350 μL/s (T 62.5s). scale bar: 40 μm. **C. D**. Amoebae adhering to non-coated glass (blue curve) or FN-coated glass (red curve). **C**. The percentage of bound cells as a function of the flow rate. The number of cells was counted in every 10^th^ image of the video, and the mean rupture force was determined from five independent runs on non-coated glass (n cells = 181) and two runs on FN-coated glass (n cells = 37). **D**. The amoebae’s adhesion forces were calculated from the friction force when the interaction with the glass surface was broken. Amoeba were considered as half-spheres with a mean diameter of 30 μm on FN-coated glass and 15 μm on non-coated glass. Differences were analyzed using an unpaired t-test (p = 0.0008, ***). Videos of two representative runs on each substrate are provided as supplementary information (S1 Video).

To determine whether *E*. *histolytica*’s binding force depends on the composition of the substrate (i.e. FN-coated glass vs. non-coated glass), we studied trophozoites seeded in a laminar flow chamber (Fig 1A and S1 Video). The trophozoites were submitted to an increasing buffer flow rate (0–640 μL/s) and imaged for cell counting (Fig 1B). Half of the cells released at 200 μL/s on non-coated glass and at 500 μL/s on FN-coated glass (Fig 1C). To estimate the cells’ binding force to the substrate, we converted the flow rate by considering the physical fluid drag (see the Methods). The median frictional force at cell release was 7.4 nN on non-coated glass and 18.1 nN on FN-coated glass (Fig 1D). Hence, the presence of FN increased the adherence strength of *E*. *histolytica* by a factor of 2.4, compared with non-treated glass.

### The number of *Entamoeba histolytica* with adhesion plates or podosomes is higher on FN

**Fig 2:**
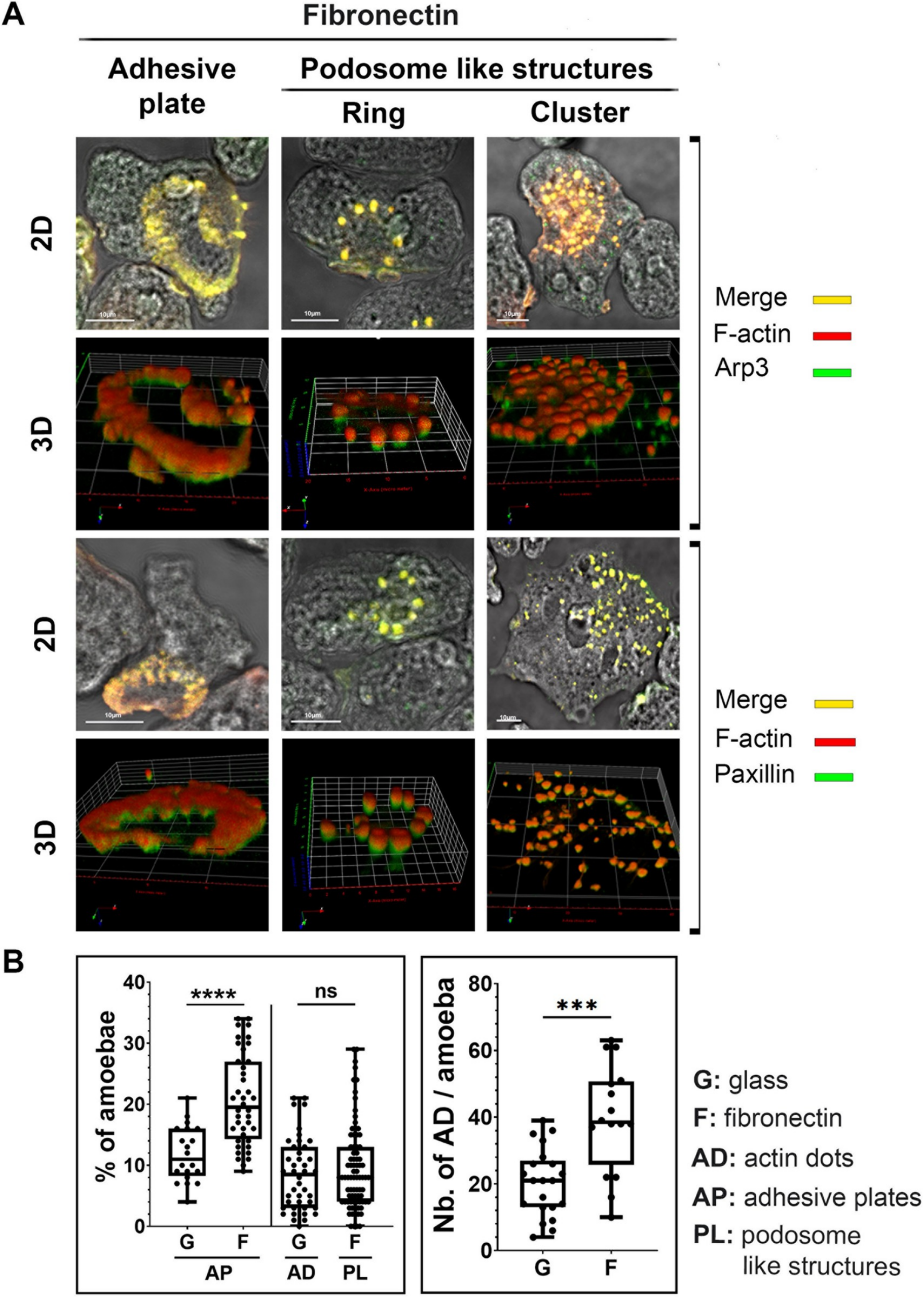
Morphological diversity of F-actin-containing adhesive structures formed by *E*. *histolytica* on an FN substrate. **A.** Actin structures found within amoeba adhering to FN-coated or non-coated glass. Fixed amoebae were labelled for F-actin (red) and Arp3 (green) in the first and second panels and for F-actin (red) and paxillin (green) in the third and fourth panels. The first and third rows correspond to two-dimensional confocal microscopy images of the merging of brightfield and the projection of z-stack fluorescent images. The Pearson’s coefficients for the correlation between F-actin and Arp3 in each representative structure through the Z-stack was 0.67 for the adhesion plate (AP), 0.90 for the podosome-like (PL) structure in a ring, and 0.76 for PL structure in a cluster; for F-actin and paxillin was 0.79 for AP, 0.73 for PL structure in a ring and 0.87 for PL structure in a cluster. The second and fourth rows show the three-dimensional reconstructions of a z-stack of images of the above structures. The xy axis (in red) indicates the ventral side of the cell adhering to the substrate. **B**. Quantification of the F-actin structures present in *E*. *histolytica* seeded on non-coated glass or FN-coated glass. The proportions of amoebae showing adhesive plates (APs), rings of dots (RDs), and clusters of dots (CDs) are shown. The left panel indicates the proportion of amoeba containing APs and actin dots (ACs) on non-coated glass (G) and the proportion of amoeba containing APs and PLs on FN-coated glass (F). The percentages of amoeba showing an actin structure in one image were expressed in a box plot: n = 113 cells on non-coated glass and n = 145 cells on FN-coated glass; n = 503 dots on non-coated glass and n = 1145 dots on FN-coated glass. The results of Mann-Whitney tests are indicated. ns: not significant. ****P<0.0001; ***P<0.001.

To analyse the effects of FN on amoebic adhesion structures, we compared the two substrates in terms of the cellular distribution of adhesion-related proteins in seeded trophozoites. Podosomes are known to contain Arp2/3 and paxillin, and the spatial organization of the podosomes can vary. We used dual fluorescence labelling and confocal microscopy to examine the localization of F-actin and its spatial relationship with Arp3 and paxillin present in amoebic adhesion structures on FN-coated glass. As expected, the structures contained Arp3 and paxillin localized with F-actin (Fig 2A). Three-dimensional image reconstruction of the focal planes showed that F-actin covered paxillin and Arp3 within adhesive structures close to the substrate (Fig 2A). We observed that podosome-like structures in amoeba could be distributed in rings or in clusters (Fig 2A). Next, we compared the number of adhesive structures per cell formed on non-coated glass vs. FN-coated glass (Fig 2B). The number of trophozoites with adhesive plates was higher on FN- coated glass than on non-coated glass-seeded, whereas similar numbers of amoeba with actin dot structures were seen on the two substrates. However, on FN-coated glass, the clusters had twice the number of dots than on non-coated glass (Fig 2B). These data show that an FN-rich substrate is associated with a greater number of adherent amoeba, and of numbers of adhesive plates and podosome-like structures per amoeba.

### The impact of inhibitors of cytoskeletal dynamics on *Entamoeba histolytica*’s adhesive structures

**Fig 3:**
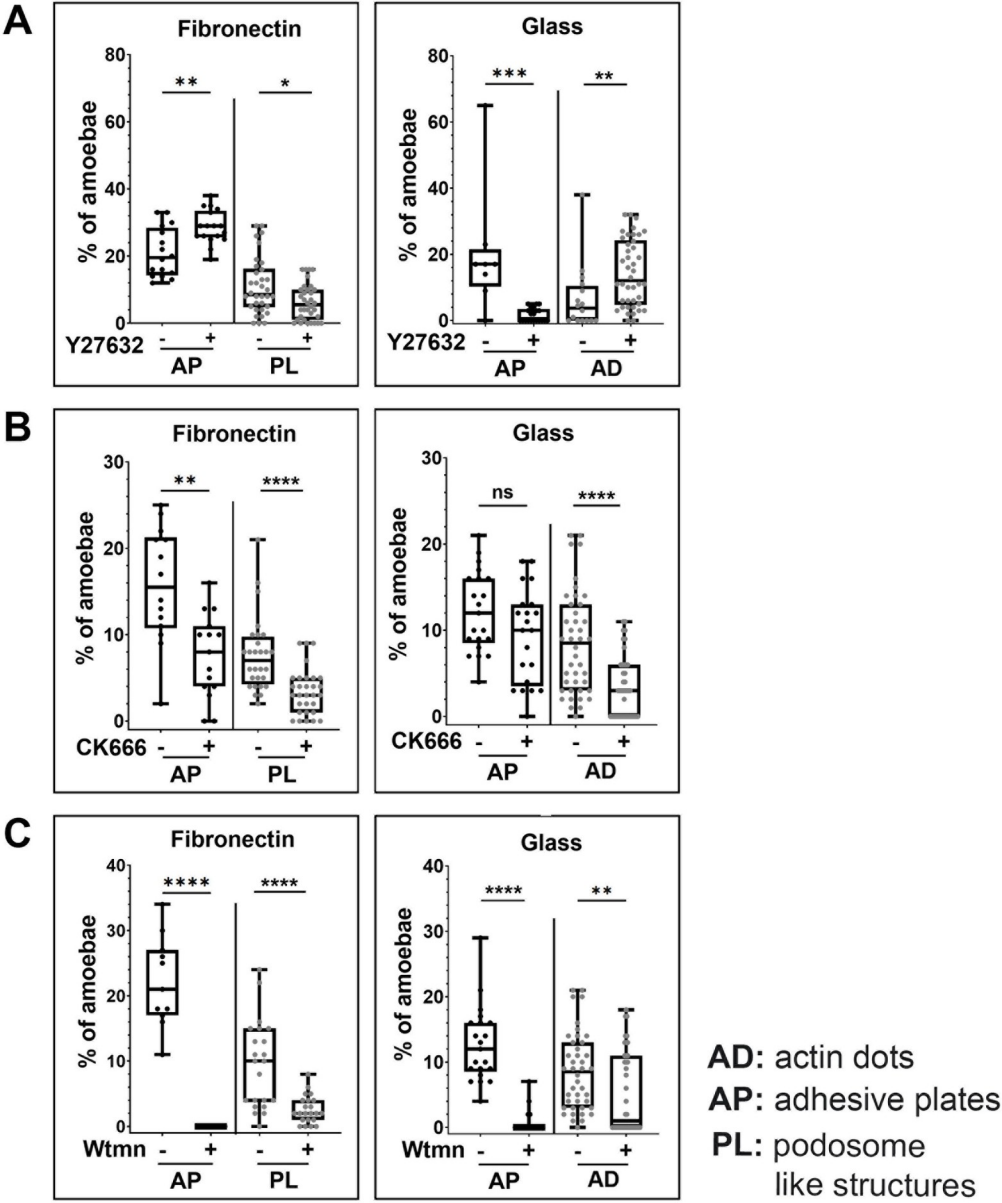
Differential effects of inhibitors (Y27632, CK666 and wortmannin) on the F-actin adhesive structures formed by *E*. *histolytica*. Amoebae migrating on non-coated glass or FN-coated glass were treated with the following compounds: (**A**) 5 μM Y27632 for 4 h, (**B**) 20 μM CK666 for 2 h, and (**C**) 3 μM wortmannin for 2 h. The percentage of amoeba per image containing F-actin labelled structures (such as an adhesive plate (AP), an actin dot (AD) and a podosome-like structure (PL)) was plotted for each condition. On FN: with or without Y27632, n = 17 cells for each condition; with CK666, n = 14 cells; without CK666, n = 15 cells; with Wtmn, n = 11 cells; without Wtmn, n = 42 cells. On non-coated glass: with Y27632, n = 21 cells; without Y27632, n = 8; with or without CK666, n = 21 cells; without Wtmn, n = 20 cells; with Wtmn, n = 21 cells. N = 4 experiments. (**D**) Volume and sphericity of dots present in *E*. *histolytica* as a function of the substrate composition and the drug treatment. For cells seeded on non-coated glass: control, n = 503 dots; Y27632, n = 389 dots; CK666, n = 273 dots; Wtmn, n = 341 dots. For cells seeded on FN-coated glass: control, n = 1265 dots; Y27632 n = 504 dots; CK666, n = 432 dots; Wtmn, n = 522 dots. Mann-Whitney test: ns: not significant. ****P<0.0001; ***P<0.001, **P<0.01, *P<0.1.

To gain insight into the nature of structural changes in adhesive structures, we incubated trophozoites with specific inhibitors of proteins involved in forming podosomes: Y27632, CK666, and wortmannin (Wtmn) respectively target ROCKs, the assembly of the Arp2/3 protein complex, and phosphatidylinositol-3-phosphate kinases (Pi3Ks) (S1 Fig). Our data suggested that in *E*. *histolytica*, the signalling pathway involving ROCK determines the formation of adhesive plates and podosomes. For cells seeded on FN-coated glass, treatment with Y27632 was associated with a greater percentage of amoebae presenting adhesion plates and a lower percentage of amoebae showing podosomes (Fig 3A left panel). In contrast, for cells seeded on non-coated glass, treatment with Y27632 was associated with a much lower percentage of amoeba with adhesion plates and a significantly greater percentage of amoebae presenting dots (Fig 3A right panel). Hence, opposite phenotypes were observed on the two substrates. This substrate dependence of the formation of adhesive structures was abolished by targeting Arp2/3 or Pi3K activities: in the presence of CK666 or Wtmn, the percentage of amoebae containing actin structures was significantly lower on the two substrates (Fig 3B and 3C). Our results data suggest that the dynamics of adhesive structures in *E*. *histolytica* are regulated in two ways: the first depends on ROCKs and the substrate’s chemical composition, whereas the second depends on the Arp2/3 complex and Pi3K but not on the substrate’s chemical composition.

### *Entamoeba histolytica* loaded on FN display an F-actin dorsum and become fan-shaped

**Fig 4:**
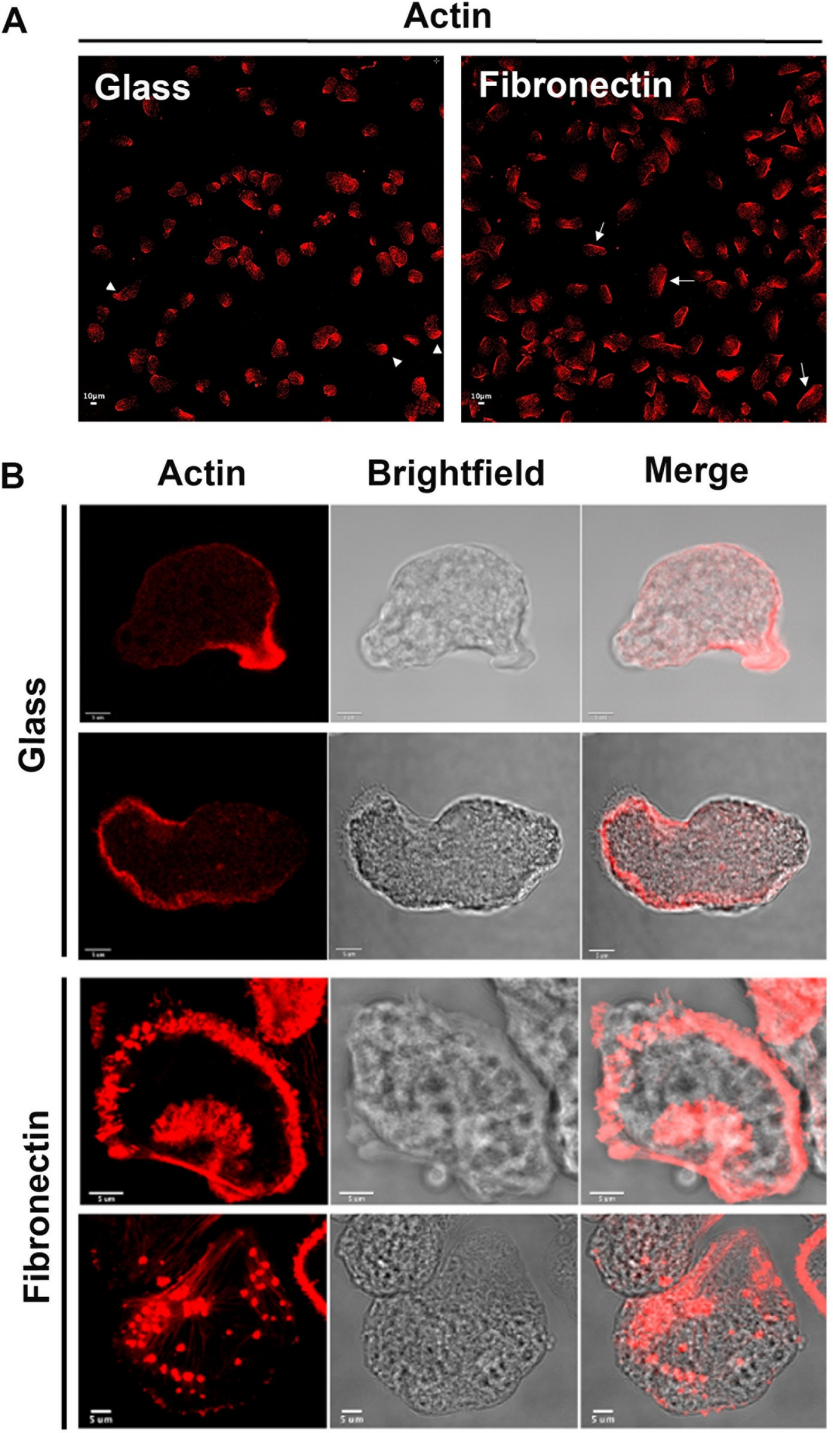
*E*. *histolytica* adopts a fan-shape morphology on a FN substrate. Confocal micrographs of amoebae loaded on FN-coated glass or non-coated glass for 1 h and labelled for F-actin. (**A**) Amoebae on non-coated glass (left) and on FN-coated glass (right). White arrowheads and white arrows indicate examples of areas of high F-actin density. A quantification of cell actin fluorescence in the image did not reveal a difference between the two conditions (S2 Fig). Scale bar: 10 μm (**B**) Examples of cells with elongated shape, showing a higher F-actin concentration on the smallest side of the cell (first row) or a reinforcement of cortical F-actin (second row). Examples of cells with a fan-like shape, showing F-actin fibres as a spine on the broader side that could radiate into the cell like the spokes of a bicycle wheel, at the leading edge (third row) and in multiple dots (fourth row). Scale bar: 5 μm.

Force-generating filamentous actin networks have important roles in cell motility [27]. The above-described changes in adhesive structures prompted us to look at how the spatial localization of actin microfilaments in amoebae changed as a function of the substrate. We observed the overall localization of F-actin in trophozoites in a wide field of view with a confocal microscope (Fig 4). Trophozoites on non-coated glass were predominantly elongated, with a high F-actin content at the rear of the cell and beneath the cell cortex. In contrast, *E*. *histolytica* bound to FN often presented a long, very straight dorsum with a high actin filament content at the rear, and a broad, fan-shaped edge at the front (Fig 4A and 4B). Fluorescence measurements indicated that the amoeba’s overall F-actin levels were higher on fibronectin (S2 Fig). The differences in F-actin level and distribution and cell shape suggest that non-coated glass and FN engage actin cytoskeletal dynamics in different ways, which in turn induces the morphological changes seen in *E*. *histolytica*.

### The morphodynamics of *Entamoeba histolytica* depend on the substrate

**Fig 5:**
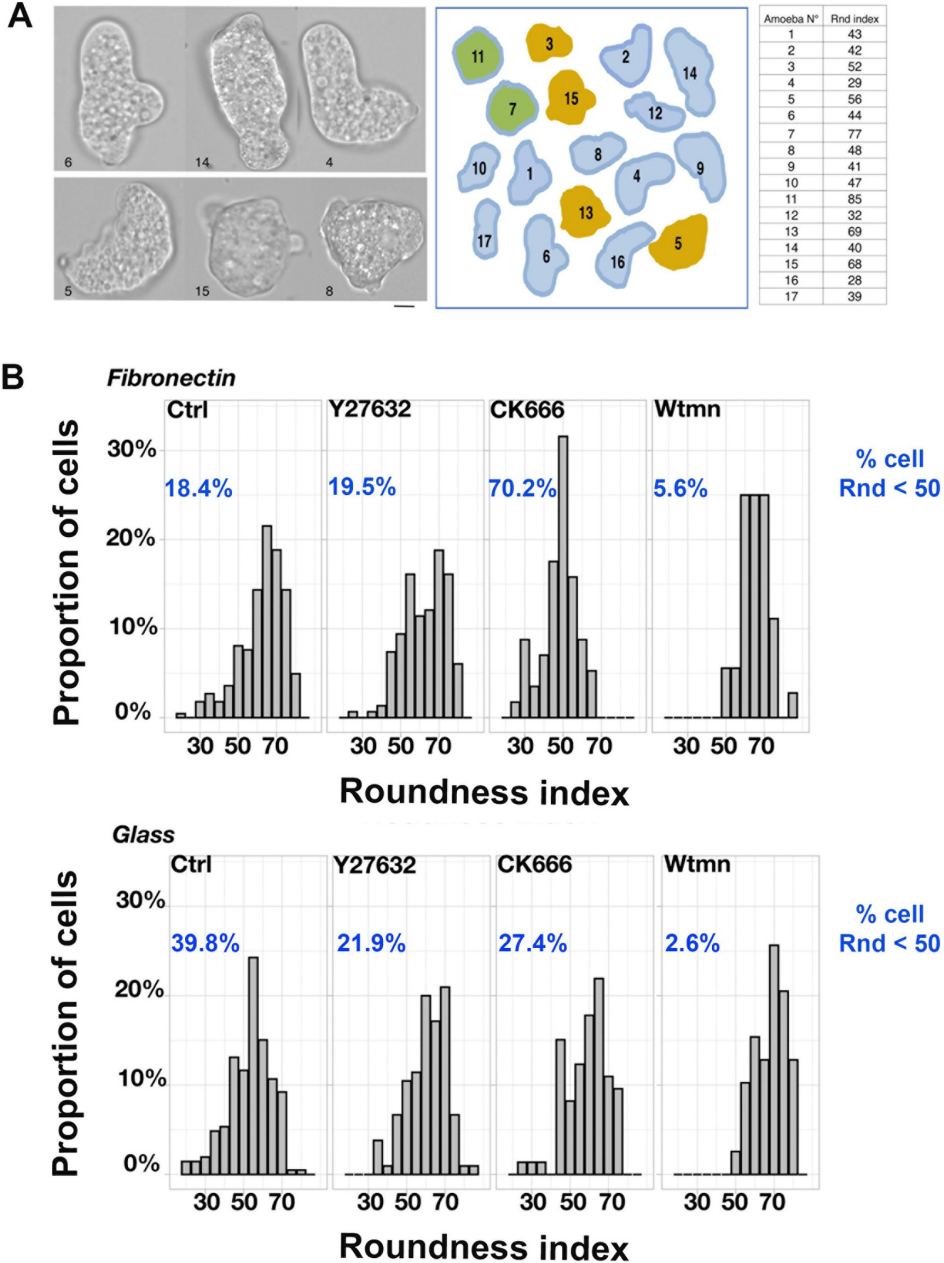
On FN, the amoeba lengthens in the presence of CK666. **A.** Examples of micrographs from six randomly selected motile trophozoites (left panel). After the segmentation of 17 cells, the trophozoites were contoured manually (middle panel) and the roundness index (Rnd) was determined (right panel). Rnd ranged from 28% to 85%. **B.** Distribution of Rnd values for cells migrating on the two substrates in the presence or absence of the inhibitors. On FN: control, n = 223 cells; with Y27632, n = 149 cells; with CK666, n = 36 cells; with Wtmn, n = 36 cells. On non-coated glass (G): control, n = 206 cells; with Y27632, n = 105 cells; with CK666, n = 73 cells; with Wtmn, n = 39 cells.

We next look at whether the substrate composition influences the morphodynamics of migrating amoebae. To this end, we quantified the mean roundness index (Rnd) of each cell on videos (Fig 5). An active-contours method was used to segment the cells [28]. Rnd ranges from a straight line (Rnd = 0) to a perfect circle (Rnd = 100). Examples of *E*. *histolytica* morphotypes and associated Rnd values (ranging from 28 Rnd for the most elongated trophozoites to 85 Rnd for the most round) are shown in Fig 5A. Next, to estimate the shape fluctuation of motile amoeba population, we calculated the distribution of the cells’ Rnd values (Fig 5B, control samples). Amoebae migrating on FN-coated glass and those migrating on non-coated glass differed significantly with regard to the Rnd (p = 0.00049), which ranged from 35 to 85 and from 20 to 70, respectively. The proportion of cells with a Rnd below 50 was twice as high on non-coated glass (39.8%) than on FN-coated glass (18.4%). These data indicate that amoebae moving on FN-coated glass are rounder than those moving on non-coated glass. We next investigated the impact of inhibitors of podosome dynamics on the shape distribution of migrating trophozoites. For trophozoites on FN-coated glass, treatment with Y27632 only slightly modified the distribution of Rnd values. Treatment with Wtmn led to a lower proportion of elongated cells, whereas treatment with CK666 led to a higher proportion of elongated cells; in the latter case, 70% of the cells had an Rnd below 50 (Fig 5B). The inhibitors had less impact on the morphological variables of trophozoites on non-coated glass. The proportion of elongated cells was lower after treatment with any of the three inhibitors, with a mean Rnd below 50 for the whole population (Fig 5B). We infer that (i) the morphodynamics of *E*. *histolytica* on FN-coated glass are finely regulated and involve Pi3K and the Arp2/3 complex in distinct signalling pathways, and (ii) the transition from an elongated cell to a round shape is enhanced by the activity of the Arp2/3 complex. Taken as a whole, our data suggest that the changes in *E*. *histolytica*’s shape depend on the distribution of the actin cytoskeleton, which is influenced by various signalling pathways (including ROCKs, Pi3Ks and Arp2/3) as a function of the substrate composition.

### *Entamoeba histolytica* migrates more slowly on FN than on non-coated glass

**Fig 6:**
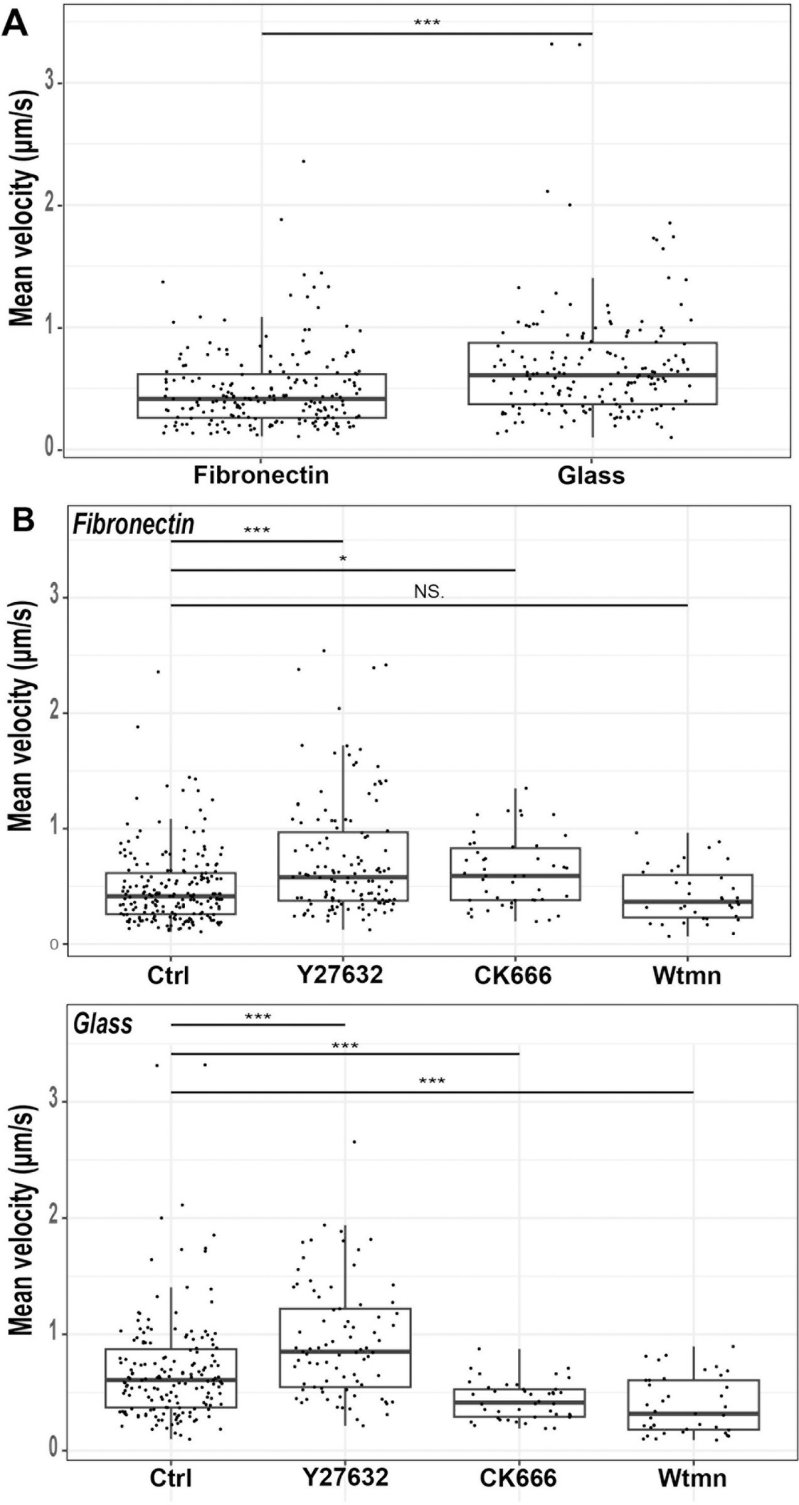
*E*. *histolytica* moves faster on non-coated glass than on FN, and longitudinal movements are faster than lateral movements. The mean velocity of trophozoites fluorescently labelled and loaded on non-coated glass (**A, C**) or FN-coated glass (**A, B**) in the presence (**B, C**) or absence (**A**) of the inhibitors Y27632, CK666 and Wtmn. Images were acquired in real time. Using Icy software, the cells were segmented, and the velocity of each cell was averaged over the whole recording. On FN: control, n = 223 cells; with Y27632, n = 249 cells; with CK666, 357 cells; with Wtmn, n = 36 cells. On non-coated glass: control, n = 206 cells; with Y27632, n = 105 cells; with CK666, n = 73 cells; with Wtmn, n = 39 cells. T-test: ns: not significant. ****P<0.0001; ***P<0.001, **P<0.01, *P<0.1.

Cell migration velocity depends on cell shape, the rates of adhesion and de-adhesion, and on the strength of adhesion to the substrate. Here, we found that *E*. *histolytica* cells migrated more slowly on FN-coated glass than on non-coated glass (Fig 6A), which suggests that the parasite adheres strongly to FN and does not have fast adhesion and deadhesion rates. We then tested the effect of compounds specifically inhibiting the ROCK, Arp2/3, or Pi3K activities (S1 Fig) on the speed of the trophozoites. The velocity of the amoeba’s displacement on both substrates was higher in the presence of Y27632. These findings suggest that ROCK downregulates *E*. *histolytica* motility and that myosin light chain phosphorylation reduces the parasite’s velocity (Fig 6B). In the presence of CK666, the speed of displacement was higher on FN-coated glass but lower on non-coated glass, suggesting that Arp2/3 activities are stimulated to a different extent on FN vs. non-coated glass. Treatment with Wtmn inhibited migration on both FN and non-coated glass (Fig 6B and 6C), indicating that the pathway inducing *E*. *histolytica* migration on both substrates requires Pi3K activities.

### On FN, fan-shaped amoebae migrate laterally

**Fig 7:**
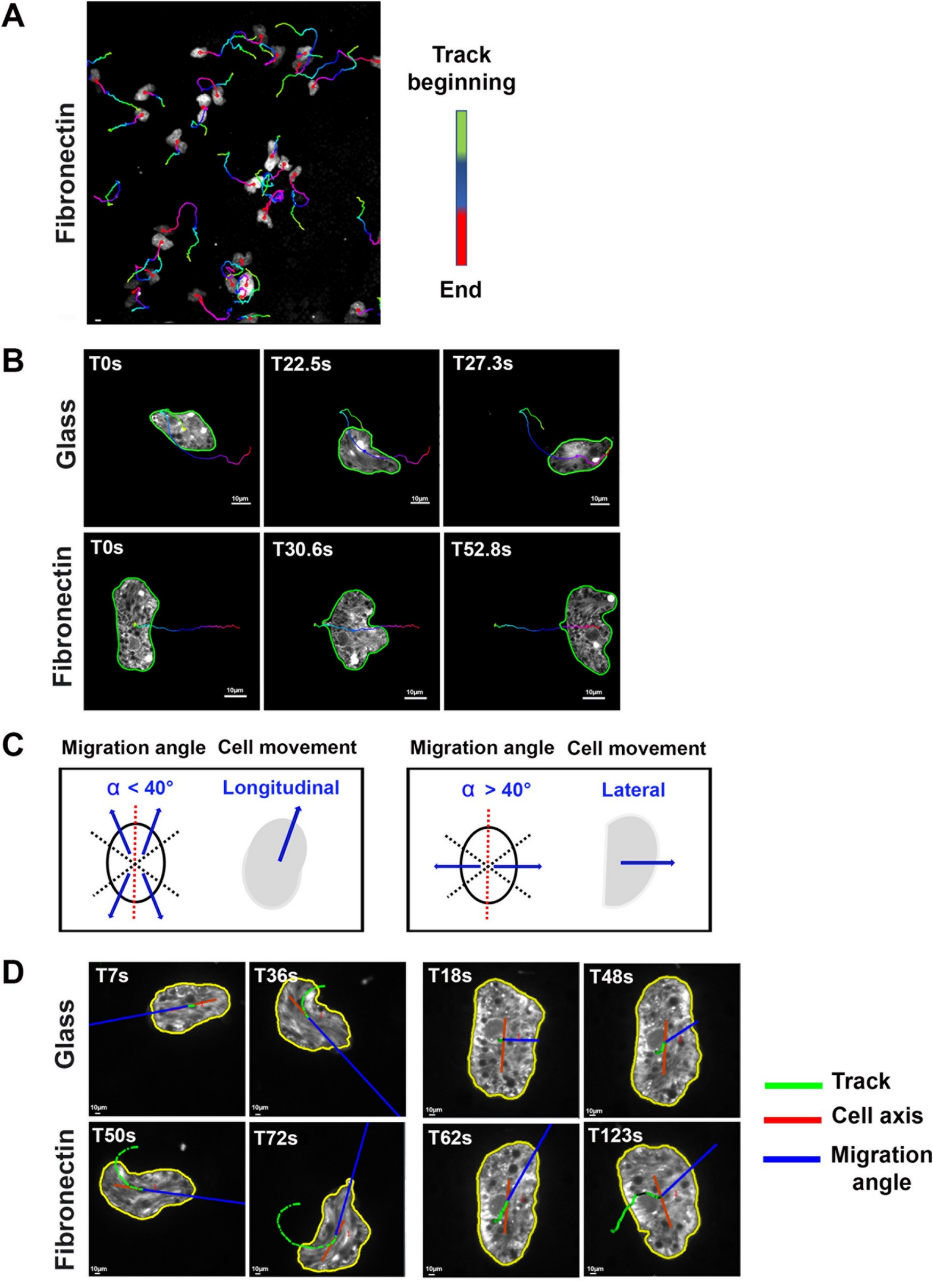
Fan-shaped *E*. *histolytica* exhibits a lateral migration mode. **A.** Trajectories of cell displacements. Trophozoites fluorescently labelled with a red cell tracker and migrating on FN-coated glass were imaged at 300 ms intervals for 3 min and segmented with the Active Contours plugin. The displacement trajectories were processed with the Track Manager in Icy software. Representative video: Sup. Data 2) **B.** Individual segmented cells. An elongated cell moving in a winding trajectory in the same direction as the cell axis (upper row). A fan-shaped cell moving in a straight path perpendicular to the cell axis (bottom row). **C.** A schematic representation of the correlation between the cell migration angle and the type of movement. **D.** Examples of *E*. *histolytica*’s migration modes. A trophozoite with the displacement angle mostly below 40° at different times during longitudinal movement on non-coated glass. A trophozoite with the displacement angle mostly above 40° during characteristic lateral movement on FN-coated glass. Representative video: S3 Video.

To determine the impact of changes in cell shape and in adhesive structures on amoeba migration, we tracked the trophozoites’ trajectories on FN-coated glass and non-coated glass (Fig 7A and 7B and S2 Video) and analyzed the angle between the migration direction and the cell axis (see Methods). Most of the trophozoites’ trajectories on FN-coated glass were lateral to the longest axis of the cell body (Fig 7A and 7B). In contrast, the trajectories of trophozoites migrating on non-coated glass were along the axis of the cell body (Fig 7B). Longitudinal movements as those with an angle below 40° and lateral movements as those with an angle of 40° or more (Fig 7C and 7D and S3 Video). The ratio between longitudinal and lateral displacements for each trophozoite indicated that (i) on FN-coated glass, the trajectories of fan-shaped cells were lateral to the cell axis, and (ii) on non-coated glass, the trajectories of elongated cells were longitudinal to the cell axis, displaying classical amoeboid movement (S3 Video).The data indicate that on FN-coated glass, the trophozoites produce a large, cytoplasmic protrusion containing podosome-like structures constituting the leading edge of the cell moving laterally.

### Displacements of fan-shaped *Entamoeba histolytica* on FN are not pressure-driven

**Fig 8:**
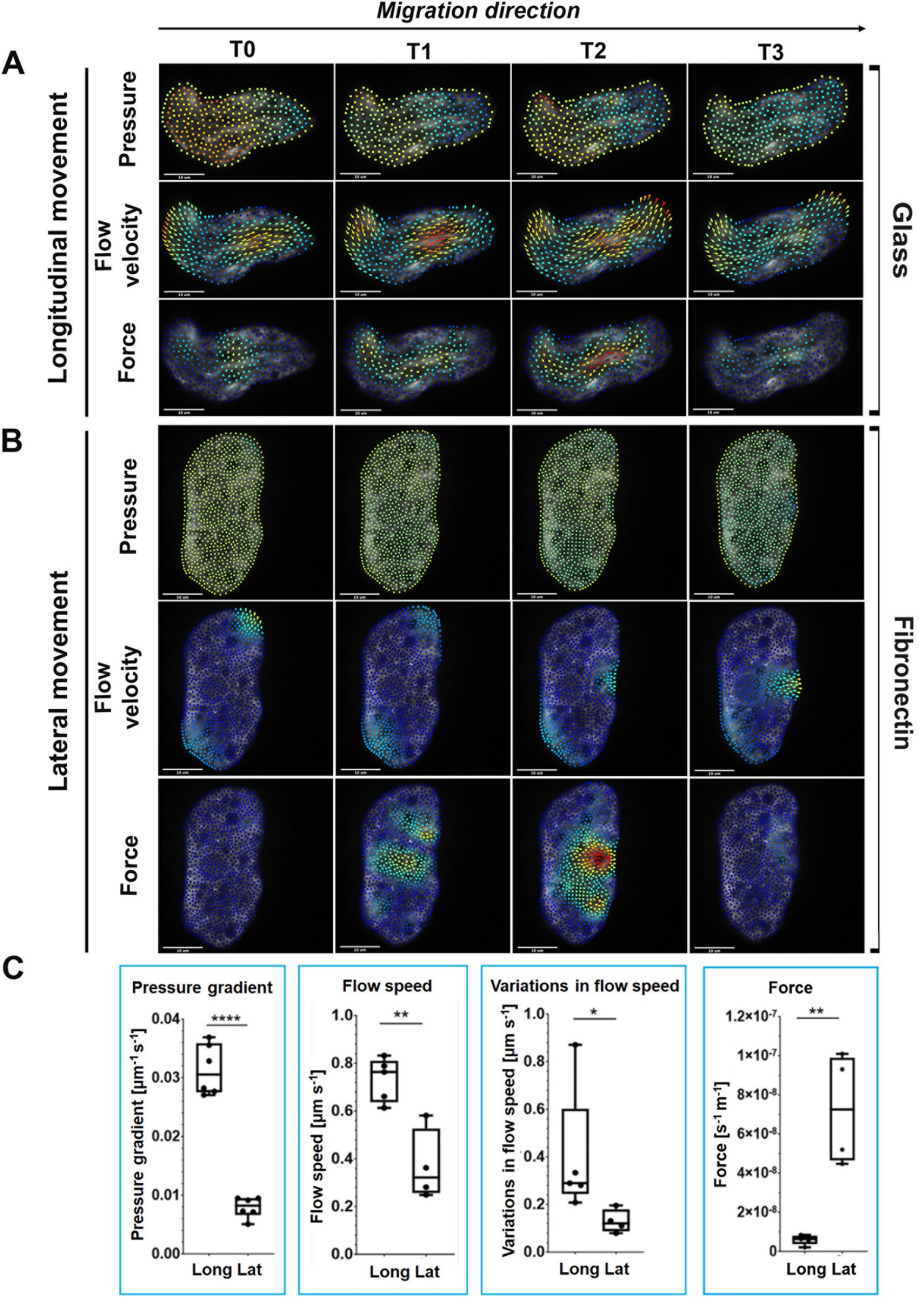
The lateral movement of fan-shaped amoeboid cells is not pressure-driven. Measurements of pressure, velocity, and force for trophozoites on **(A)** non-coated glass, and **(B)** FN-coated glass. The snapshots displayed were rendered using Bioflow Display. The values are laid on top of the raw images at different time points. The force and pressure are measured relative to the cell’s viscosity constant. The colour scale differs for A and B: blue low to red high. Pressure [s^-1^]: A (0–4.1), B (0–2.1); velocity [μm s^-1^]: A (0–1.9), B (0–1.1); force [m^-1^s^-1^]: A (0–1.7⋅10^−8^), B (0–19.3⋅10^−8^). Longitudinal movement: T1: 220 ms, T2: 330 ms, T3: 440 ms. Lateral movement: T1: 210ms, T2: 315ms, T3: 420 ms. **C.** Four different quantitative variables that differ when comparing the two types of movement (see the Materials and Methods). “Pressure gradient” refers to the difference in pressure values within a cell, “flow speed” is the magnitude of the flow velocity, “variations” refers to the variance in time and is meant to quantify how much the velocity changes, and “force” refers to the magnitude of the force vector. Longitudinal movements appeared to be faster and pressure-driven, whereas lateral movements rely more on force. Unequal-variances *t*-test. *: 0.05; **: 0.01; ***: 0.001; ****: 0.0001 (n = 5,6 per condition).

Given that the difference in adhesion structures displayed by amoebae on FN-coated vs. non-coated glass leads to different migration strategies and morphologies, we next sought to establish whether the intracellular mechanics also differed (as suggested by the results of the inhibitor experiments). To this end, we quantified pressure, forces, and cytoplasmic flow (see the Methods section on image quantification of intracellular biophysical variables) inside freely moving trophozoites displaying either longitudinal or lateral motion. Longitudinal amoeboid movement on non-coated glass is based on the formation of blebs and pseudopodia. We observed that this mode of migration involves the formation of pressure gradients across the cell (in the direction of motion), which help to fill the growing protrusions (Fig 8A and S4 Video). In contrast, the lateral movement of fan-shaped amoebae on FN-coated glass was not driven by pressure to the same extent (Fig 8B and S5 Video): the pressure gradients in the cell were smaller and more localized (Fig 8B). Instead, amoebae moving laterally relied more on intracellular forces (Fig 8C). In general, pressure-driven movement led to faster cytoplasmic streaming (Fig 8C), whereas force-driven movement was slower but more constant (Fig 8C). In agreement with our migration speed experiments (Fig 6), the faster overall cytoplasmic streaming in cells moving longitudinally translated into faster migration. Taken as a whole, these data show that adaptation of the migration mode to the substrate involves changes in actin cytoskeleton dynamics, which in turn lead to changes in cell shape and in the biophysical variables needed to drive cell movement (e.g. pressure gradients and forces).

## Discussion

**Fig 9:**
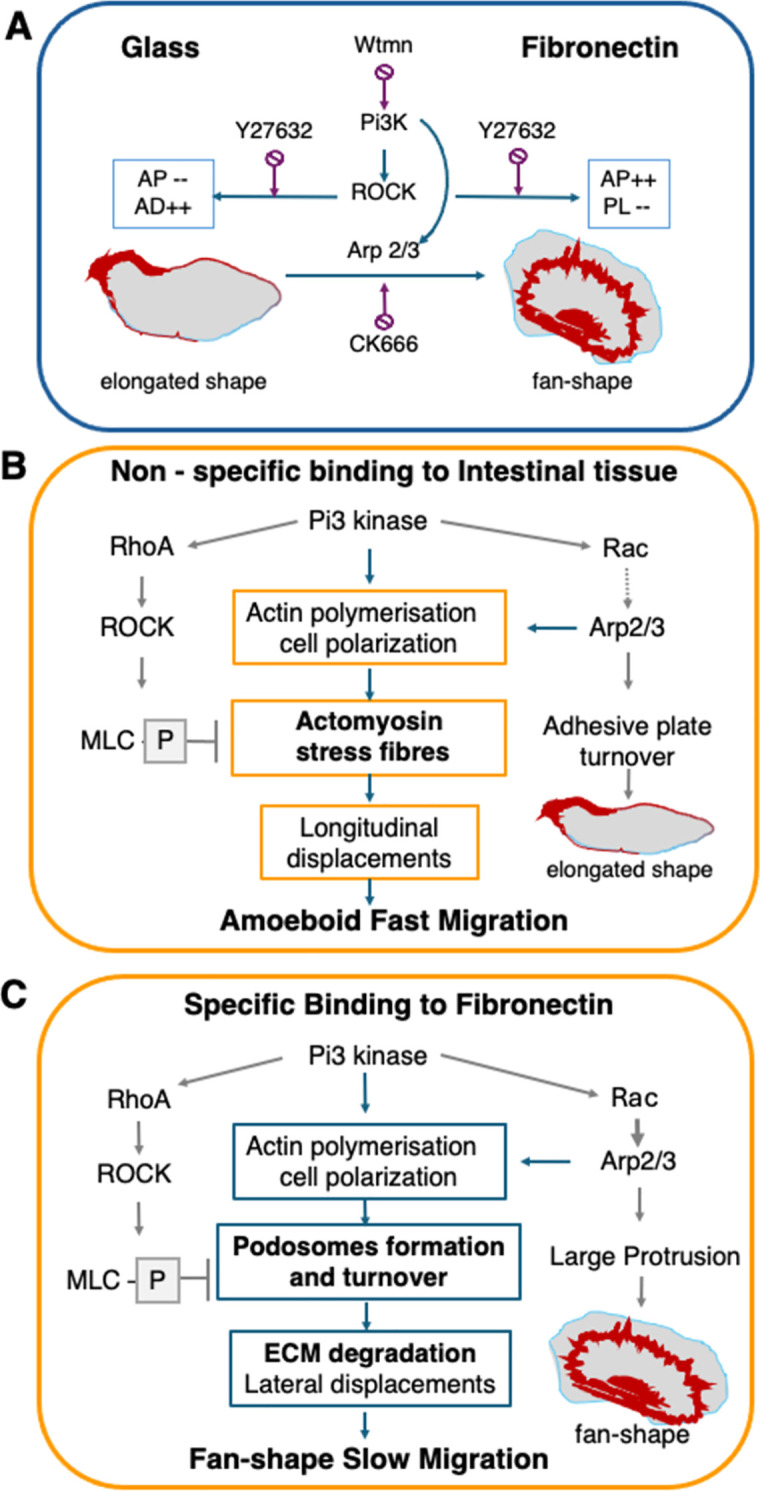
Models of signalling features during the movement of *Entamoeba histolytica*. Three scenarios are summarized by considering possible pathways for the intracellular dynamics of trophozoites subjected to various stimuli. **A.** The diagram represents data from our *in vitro* experiments on trophozoites loaded onto non-coated glass or FN-coated glass. The activity of Pi3 kinase (Pi3K) is the main regulator, independent of the nature of the substrate. Pi3K can be inhibited by wortmannin (Wtmn), leading to a loss of morphology and adhesive structures and less cell migration. Pi3K is known to act selectively on small GTPases (see S1 Fig). Next, phosphoinositides activate ROCK in a substrate-dependent manner. ROCK is responsible for the formation of adhesion plaques (APs) and actin dots (ADs) on non-coated glass, and podosome-like structures (PLs) on FN-coated glass. In the presence of Y27632, the balance of these structures is modified (shown inside the square). Through various effectors, Pi3K signalling also activates Arp2/3 complex assembly, which is responsible for a rapid change towards a fan-shaped cell morphology on FN-coated glass. Inhibition of Arp2/3 by CK666 is associated with a lower proportion of fan-shaped cells. **B.** A working hypothesis for *E*. *histolytica*’s movements in areas of tissue lacking FN. We hypothesize that elongated trophozoites move primarily in an amoeboid manner; they are able to penetrate a “soft” environment (such as mucus and fibrillar collagen I in the ECM), as previously observed in a human intestinal explant model. The actomyosin cytoskeleton is essential for this activity because it generates the intracellular pressure gradient that pushes the cell body forward. **C.** A working hypothesis for *E*. *histolytica*’s movements in areas of tissue rich in FN. Trophozoites can specifically bind to a FN receptor, which triggers a signalling pathway responsible for morphological changes to a fan-shaped cell and the formation of podosomes, which attach the amoeba strongly to FN-coated glass and reduce the migration speed. Podosomes have an impact on *E*. *histolytica’s* invasion in the intestine because they are FN degradation sites thought to facilitate the destruction of the colonic architecture and thus allow the parasite’s progression into the tissues.

Our major finding is that *E*. *histolytica* uses at least two different modes of migration: a classical, longitudinal, amoeboid movement based on the formation of pseudopodia, and a lateral migration based on a large, cytoplasmic protrusion. Fan-shaped cells form and migrate spontaneously within a population of trophozoites loaded onto an FN substrate, and these processes are sustained by the remodelling of the F-actin-rich cytoskeleton. Our results are reminiscent of those reported for *Dictyostelium discoideum* in which the motility mode depended on genetic factors, developmental conditions, and intracellular signalling [10]. Indeed, studies of *D*. *discoideum* have shown that the aggregation minus B-null mutant [29], the Glia maturation factor (GmfA) knock-out mutant [9], and a strain lacking the *axeB* gene (encoding neurofibromin, a small Ras GTPase activator) display a fan-shaped cell migration mode [10,30]. Interestingly, GmfA is an actin depolymerising factor that inactivates Arp2/3 complex formation. Accordingly, the *gmfA* knock-out mutant shows a broader migration front that is riched in Arp3; this mutant migrated with higher persistence (defined as the distance between the origin and endpoints divided by the actual distance along the cell trajectory) and adhered to the substrate more strongly than wild-type cells [9]. The phenotype of fan-shaped *E*. *histolytica* correlated with that of gmfA-null *D*. *discoideum*. However, a simple modification of the substrate (the addition of FN) had the same effect on *E*. *histolytica* as mutations did on *D*. *discoideum*. In both cases, changes in F-actin dynamics led to the fan-shape migration mode. The transition from amoeboid motility to fan-shaped motility has been described in terms of the actin-wave model in several cell types, including *D*. *discoideum* [10]. The waves are F-actin-rich transitory protrusions of the cell border that move along the periphery during cell displacement [31]. Fan-shaped cells are formed by actin wave nucleation and growth from amoeboid cells; the actin wave grows and eventually fills most of the cell’s ventral cortex, forming a stable fan-shape that persists until the wave breaks down spontaneously [10]. Before changing its migration mode, the cell explores the environment’s chemical and mechanical properties and modifies the ECM’s architecture (e.g., density, stiffness, dimension, and molecular composition) in response to various factors [32]. In amoeboid cells, the dynamics of the actin cytoskeleton’s retrograde flow depend on substrate’s texture and thus the shear forces needed to move the cell body forward. Motile *E*. *histolytica* are generally described as having a classic elongated shape of cell with amoeboid motility based on pseudopod extension and F-actin concentration at the cell rear, where the actomyosin motor contracts, creating an intracellular pressure gradient which pushes the cell body forward [16,18]. Our present results showed that when *E*. *histolytica* is placed on FN-coated glass, it adopts a fan-shaped morphotype, with an F-actin-rich area at the back of the cell and a perpendicular, round, broad cytoplasmic surface harbouring podosomes in a ring or in a cluster. We found that fan-shaped trophozoites move with a weak pressure gradient; this suggests that the actomyosin motor is less active and less able to push the cell body but sufficient to detach podosomes present in the broad cytoplasmic migration front. We conclude that the adhesion of *E*. *histolytica* to FN induces specific changes in the dynamics of the actin cytoskeleton, which in turn modify the morphophenotype and intracellular biophysical variables–both of which contribute to transitions between migration modes. In *D*. *discoidum*, a reduction in levels of phosphatidylinositol-4,5-bisphosphate and/or an increase in activities of small GTPases Ras/Rap modifies signalling on the actin cortex and allows morphological changes [33]. In contrast, fan-shaped cells converted to the amoeboid shape via the inhibition of Ras effectors and the restoration of directed cell migration [33]. Our data (Figs 9 and S1) are in line with these earlier reports and demonstrate that signalling pathways are orchestrated by PI3Ks during a substrate-mediated change in motility in *E*. *histolytica*. Moreover, small GTPases are known to be influence the architecture of adhesive structures induced by FN [23,34]. Our data showed that the force generated by *E*. *histolytica* plated on FN-coated glass is 2.4 times greater than that on non-coated glass. Trophozoites might adhere to non-coated glass via non-specific van der Waals and electrostatic interactions, as has been suggested for *Dictyostelium* [35,36]. On FN, trophozoites might adhere through a potential α1-integrin like receptor [25,26], although the latter’s existence in *E*. *histolytica* is subject to debate. Regardless, *E*. *histolytica* exhibits actin structures such as adhesive plates and punctate actin podosome-like structures [24,34,37]. We showed that like in mammalian cells, *E*. *histolytica* podosomes are highly dynamic microdomains located on the ventral side of the cell, where they form a ring or a cluster of dots and contain F-actin, Arp3, and paxillin. The fact that adhesion plates in *Entamoeba* contain Arp3 suggests that they are very different from mammalian focal adhesion plates, which are organized by integrin clustering upon FN binding and do not include Arp2/3 [38]. *E*. *histolytica*’s adhesion complexes do not appear to be stable and are reminiscent of transient, paxillin-containing adhesion sites in *D*. *discoideum* or in neutrophils, for which a specific integrin-ECM interaction has not been identified [39]. After determining the proportion of amoebae with at least one actin structure and the number of podosomes per cluster, we concluded that the greater adhesion force on FN is due to increase in the number of adhesion plaques and formed podosomes attaching the amoeba to the substrate. In mammalian cells, the sequence of podosome formation and dissociation has been well characterized. Briefly, Pi3K (the upstream regulator) accumulates at the site where podosome will form. Next, an actin network branched by Arp2/3 assembles [40]. An oscillatory membrane protrusion is reinforced by RHOA-ROCK activity, which leads to the recruitment of supervillin. Greater myosin II contractility induces the dissolution of the podosome [41–43]. The ROCK pathway is known to be involved in podosome maturation [44] and dynamics [41,43]. We investigated the roles of Pi3K, Arp2/3 and ROCK in the formation and plasticity of actin-rich adhesive structures in *E*. *histolytica*. Our results showed that (i) Pi3K activities are essential for forming all actin structures on both substrates, (ii) Arp2/3 activities are essential for constructing adhesion plates on FN, and actin dots and podosomes (in rings or clusters) on non-coated glass and on FN respectively, (iii) ROCK activity is involved in the dots’ plasticity but depends on the substrate. ROCK activity is essential for forming or stabilizing *E*. *histolytica* adhesion plates on non-coated glass and, in contrast, for dissolving them on FN-coated glass. Concerning the actin dots on glass, ROCK reduces their number, and for the podosomes on FN, ROCK increases their number. In the highly motile free-living *Amoeba proteus*, a hypercontracted phenotype was observed after treatment with ROCK inhibitors; the researchers suggested that inactivation of ROCK leads to activation of myosin II [45]. Intriguingly, we found that ROCK inhibition was associated with faster *E*. *histolytica* trophozoite migration on FN-coated glass. Our data prompt us to suggest two non-exclusive hypotheses regarding ROCK inhibition in the parasite: (i) an increase in myosin II activities leads to hypercontraction of the actin-myosin motor and faster migration; (ii) a lower number of smaller podosomes fails to bind effectively to the substrate, leading to faster migration. Further cellular and biochemical studies will be necessary to understand the role of ROCK during *E*. *histolytica* migration on FN-coated glass. Taken as a whole, our results show that *E*. *histolytica* cells on FN has more podosomes, which strongly enhances adhesion and induces a change in the cell’s shape and its migration mode. Podosomes have an impact on *E*. *histolytica’s* invasion in the intestine because they are FN degradation sites thought to facilitate the destruction of the colonic architecture and thus allow the parasite’s progression into the tissues [24,34]. A lower migration velocity might favour the concentration of enzyme activities within a path underneath the cell and thus penetration of the mucosa. Podosomes also have a mechanosensing function [46], so *E*. *histolytica* might be able to sense the environment and adapt its adhesion force to the stiffness of the tissue or/and mechanical constraints such as colonic peristalsis, which has been shown to facilitate the parasite tissue invasion [47]. The podosomes may allow the trophozoites to anchor strongly to FN beneath the epithelial cells and thus migrate toward the intestinal crypts, where the parasite penetrates into the tissues [13]. According to our working hypothesis (Fig 9) *E*. *histolytica* adopts an amoeboid migration mode in a “soft” environment (e.g. mucus and the collagen I fibrillar ECM network) and a fan-shaped migration mode on FN below the epithelium. In conclusion, the dynamic amoeboid *E*. *histolytica* can adjust its migration mode as a function of the interplay between the environment and the dynamics of the actin-rich cytoskeleton. Studying *Entamoeba* migration within human colon models might generate new information on the invasive process.

## Materials and Methods

### *Entamoeba histolytica* culture

Trophozoites of the *E*. *histolytica* strain HM-1: IMSS were grown overnight at 37°C in TYI-S-33 medium [48]. The amoebae were rinsed with TYI-S-33 medium devoid of serum and vitamins (TYi media) before carrying out the experiments.

### Rupture force assay

The adhesion force of *E*. *histolytica* interactions on FN-coated and non-coated glass was evaluated within a laminar flow chamber as described previously for lymphocytes [49,50]. The glass surface (RS, France) assembled to a flow chamber (Slide I^0.1^, Ibidi, Germany) was washed with 5 chamber volumes (CV = 25 μL) of sodium hydroxide (2 M, Merck-Sigma), 20 CV of water, 5 CV of sulfuric acid (2 M, Merck-Sigma), 20 CV of water, then 5 CV of hydrogen peroxide (33% Merck-Sigma), 20 CV of water then 20 CV of PBS. When described, bovine fibronectin (10 μg/mL in PBS, Merck-Sigma) was added for 1 h at room temperature and then rinsed with 20 CV of PBS. The chamber was equilibrated with TYi at 37°C then loaded with *E*. *histolytica* (10^6^ trophozoites/mL) for 15 min at 37°C. A PBS flow rate increasing from 0 to 38.4 mL/min 40 μL/s), was applied through the chamber for 115 s using a syringe pump (SP210iW, WPI) controlled by a computer, and synchronized with image acquisition (3 image/s, 1100 x 840 μm) using an inverted transmission microscope (Observer D1, Zeiss) with a 10x/ 0.3 NA objective piloted with MicroManager. The flow rate value at the cell-substrate rupture event was used to compute the dragging force releasing the trophozoite according to its shape (half sphere), size (mean diameter of adhesion surface area to glass 30 μm, thickness 15 μm) and density (mean value *p =* 1.20 kg/L). Calibration of dragging force was performed from the sedimentation rate of cells in the chamber (measured PBS density 1.0034 kg/L and dynamic viscosity, ri = 0.6998 mPa/s at 37°C) and the theoretical flow speed *v* versus wall distance according to Poiseuille solution to Navier-Stokes formalism for a Reynolds’ number below 10 characterizing a laminar flow. The Stoke’s dragging force (Fs) was expressed for a spherical object as: Fs = 6 n ri *v* R (where, *v*, R represent the dynamic viscosity, the fluid speed and the cell radius respectively). It is reduced to Fs = 3 n ri *v* R if we consider half a sphere representing the hemi-spherical adherent cell used as mean reference. The linear speed of the liquid solution *v* in the laminar flow chamber depends on the distance from the chamber walls and the flow rate through the chamber. The speed of the liquid, *v* has been calculated for every 1 μm-layer from the glass floor to the maximal height of cells, 15 μm. The dragging force on the cell has been approximated considering the averaged speed *v* from 0 to 15 μm. The dragging force at the breaking time of the *E*. *histolytica* interactions with the FN-coated or the non-coated glass when the cells is carried away in the flow, was considered as the adhesion force.

### Coating and incubation conditions

10^4^ trophozoites were loaded on 35 mm glass-bottomed imaging Ibidi dishes filled with 6 ml of TYi at 37°C. The Ibidi dishes were untreated or pre-coated with 100 μg/ml bovine fibronectin (Merck-Sigma) overnight at room temperature and then washed twice with BPS. For inhibition studies, amoebae were incubated before image acquisition with 20 μM CK666 (Sigma-Aldrich) for 2 h to inhibit ARP2/3 complex nucleation, 5 μM Y27632 (BD Bioscience) for 3 to 4 h to inhibit ROCK activities or 3 μM wortmannin (Sigma) 2 h to inhibit PI3Kinases. Incubation was performed at 37°C in GENbag anaerobic device (Biomerieux, Marcy-l’Étoile, France). These concentrations were chosen according to precedent publications for 3uM wortmannin [51], 40uM CK666 [52] and for 5uM Y27632 (this work). BD Bioscience’s guidance for use of Y27632 is at a concentration of 10 μM in mammalian cells. In our preliminary testing experiments (using 5 μM), no cell death was observed and changes in amoeba speed were significant. We therefore estimated that the concentration of 5 μM did not present a risk of off-target effects as it has been shown in other works (e.g., 40uM) [53].

### Fluorescence, confocal microscopy of fixed cells and image quantification

Immunofluorescence microscopy was performed on amoebae fixed with 4% PFA solution (ThermoFisher Scientific). The cell membranes were permeabilized with 0.05% Triton X-100 in PBS for 3 min. The slides were washed with PBS, quenched with 50 mM NH4Cl for 15 min and blocked with 2% BSA for 1 h. The cells were incubated for 2 h at RT with primary antibodies diluted in 1% BSA, rabbit polyclonal anti-paxillin (1:200) [51]or rabbit polyclonal anti-Arp3 (1:200) [52]and then rinsed with PBS and incubated for 1 h at RT with a secondary, AlexaFluor-546 labelled, goat anti-rabbit antibody diluted 1:200 in 1% BSA (Molecular Probes). To decorate filamentous actin (F-actin) the cells were incubated with phalloidin AlexaFluor-488. Coverslips were washed with BSA-free PBS and mounted with ProLong antifading reagent. Z-stacks of confocal planes (step size: 0.5 μm or 0.1 μm) were acquired with a confocal microscope (LSM700, Zeiss, Germany) and Zen software. A 40x objective, NA = 1.3, pixel size of 0.1 μm, xy resolution 0.16 μm was used to quantify the number of cells containing actin structures (adhesive plates, clusters and ring of dots) and a 63x oil objective, NA = 1.4, pixel size of 0.07 μm, resolution xy 0.177 μm to reconstruct the actin structures within the cell in three dimensions.

To number the cells containing actin structures, the images of the Z-stacks were analysed using the intensity projection plugin with the maximum projection parameters of the Icy software [54]. Then cells were annotated manually using the Region of Interest tool (ROIs) to sort the cell by the attributed forms as a polygon, a small circle and an ellipse for cells containing an adhesive plate, a cluster of dots or ring of dots, respectively. A cell containing several similar structures was annotated once, but a cell containing several different structures was annotated for each structure. Cell sorting results were exported into Excel files for data analysis. For each image, the percentages of amoebae with specific annotated forms were plotted using Prism GraphPad Software version 9.5.1, Boston, MA, USA.

### Single-cell motility assay and live-cell imaging

Trophozoites after overnight culture were labelled with Cell Tracker Red (CMTPX ThermoFisher C34554) (2.5 μM) in TYi medium for 1 h at 37°C before loading them (10^4^ cells) to raw or fibronectin-coated glass bottom Ibidi boxes. Live cell imaging experiments were conducted on an inverted microscope (Axiovert 200 M; Carl Zeiss) equipped with a spinning-disk confocal system (Ultraview VOX, Perkin Elmer) with a 10x/NA = 0.3, objective, pixel size of 1.6 μm or a 63x oil-immersed objective (plan-Apochromat; NA = 1.4), with a pixel size of 0.4 μm to analyse the morpho-dynamic parameters and the intracellular biophysics parameters respectively. The fluorescent signal (emission wavelength: 545 nm; detection bandwidth: 480–570 nm) was recorded on an electron-multiplying CCD camera (Andor EMCCD DV885). Videos were recorded for 4 min at an imaging rate of one frame per 300 ms. Time-lapse sequences were acquired at full imaging rate (i.e. no delay between individual time points) for 4 minutes. The files and their associated metadata were in a mvd2 format.

### Image quantification of cell morphodynamics parameters and angle of migration

To quantify the changes in trophozoite shape, the video movies were analysed with the Active Contours plugin of Icy [28]. The resulting cell contours were exported and stored together with the contour characteristics, including the perimeter, the position of the centre and the orientation of their main longitudinal axis. The Roundness (Rnd) is a measure of how similar to a circle the cell contour is. Rnd is defined as the ratio of the radius of the largest circle contained in the contour to that of the smallest circle containing the contour. In Icy both circles are approximated by circles centred on the centre of mass of the contour. The cell shapes could range from completely elongated to perfectly round corresponding to Rnd from 0 to 100. The Rnd of a cell represents the mean of its roundness taken on all images of the video recordings. For each substrate and pharmacological treatment, the distribution of Rnd was plotted. To determine the characteristics of trophozoite displacements, the instant cell velocity was measured using video frame pairs and the mean of instant velocity was computed over the full recording. The angle between the main cell axis and the direction of its displacement between two successive time points was measured for each frame of a movie. A displacement at an angle smaller than 40 degrees from the longitudinal axis was considered as a longitudinal movement [55]. Cells were classified as having globally longitudinal (Long) movement if at least 80% of the individual movements were longitudinal and classified as lateral (Lat) otherwise.

### Image quantification of intracellular biophysics parameters

We imaged freely moving trophozoites that were fluorescently labelled with Red Cell Tracker using a confocal microscope (see Single Cell motility assay and live imaging). The image sequences were analysed with the last version of BioFlow. This method estimates spatiotemporal maps of pressure *p*(*x*,*t*), velocity *v*(*x*,*t*), and forces *f*(*x*,*t*) inside the cells in a non-invasive way. It does so by following the movement of the image intensity through the image sequences [56] while constraining it to fulfil the equations of continuum mechanics. The extracted values are normalized by the viscosity constant of the cell. We ran the method on two cell populations: trophozoites on non-coated glass, and trophozoites on fibronectin. While the differences between the spatiotemporal maps were visible to the naked eye, we applied a quantitative comparison to each cell individually and finally averaged over the population. To this end, we devised four measures. The first measure is the pressure gradient across the cell, in the direction of movement. If the front and back of the cell (defined by the direction of motion) are at *x*_*f*_∈*Ω* and *x*_*b*_∈*Ω* then we calculated $\left(p(x_{b})-p(x_{f})\right)/\left\|x_{f}-x_{b}\right\|$, where *Ω*⊂*R*^2^ is the domain of the cell. This was collected over multiple time points. We call the second measure “flow speed”. It is the average over space and time of the norm of the velocity. More specifically, $\overset{´}{v}=\sum_{k=1}^{T}\bar{v}(t_{k})/T$, where $\bar{v}(t_{k})=\int_{\Omega }\left\|v(x,t_{k})\right\|/\left|\Omega \right|$ is the spatial average, and *T* is the number of temporal points. The third measure is the temporal variation in flow speed as calculated by the standard deviation (over time) of the average (over space) flow speed: $\overset{´}{v}=\sqrt{\sum_{k=1}^{T}{\left(\bar{v}(t_{k})-\overset{´}{v}\right)}^{2}/\left(T-1\right)}$. The last measure is the average force generated by the cell: $\overset{´}{f}=\sum_{k=1}^{T}\int_{\Omega }\left\|f(x,t_{k})\right\|/\left(T|\Omega |\right)$. The p-values were derived from unequal-variances t-tests.

### Statistics

We analysed the adhesion of *E*. *histolytica* on fibronectin versus glass with an unpaired t-test (p = 0.0008, ***) using the GraphPad Prism 8.0 software for Windows (La Jolla, CA, USA) The measurements for controls or inhibitor-treated cells were exported as Excel files for data analysis and compared using Prism GraphPad Software version 9.1.2, using a non-parametric unpaired Mann-Whitney statistical test. It is a nonparametric test that compares two unpaired sets of data significantly different when the *p* value is less than 0.05 (**** extremely significant *P* < 0.0001, *** extremely significant 0.0001 < *P* < 0.001, ** very significant 0.001 < *P* < 0.01, * significant 0.0.1 < *P* < 0.05 and ns not significant ≥ 0.05).

## Supporting information

S1 Fig Brief diagram showing the pathways blocked by protein inhibitors involved in mammalian cell motility. Phosphoinositide 3-kinases (PI3Ks) phosphorylate the membrane lipid phosphatidylinositol-4,5-bisphosphate [PI(4,5)P2] to generate the lipid second messenger phosphatidylinositol-3,4,5-trisphosphate (PIP3) that controls the actin cytoskeleton mainly through activating the guanine exchange factors of small GTPases including Rac1 and RhoA, which govern cell adhesion dynamics and motility. The two most thoroughly characterized types of cell migration are: (i) the amoeboid mode driven by the contractile actomyosin cortex of the cell (left side of the figure). Following RhoA activation of ROCK activities myosin light chain became phosphorylated and the actomyosin functions regulates F-actin stress fibre dynamics and focal adhesions. (ii) the mesenchymal adhesion-dependent migration mode (right side of the figure). Depending on the activity of Rac1 on various intermediate effectors, the Arp2/3 complex nucleates and branches actin filaments forming networks of fine sheet-like lamellipodial protrusions at the cell leading edge and adhesive structures as podosomes (P). Wortmannin (Wtmn) inhibit Pi3Ks, Y27632 inhibit ROCK, and CK666 inhibit Arp2/3 complex assembly. See references in the main text.
(TIFF)

S2 Fig F-Actin fluorescence image quantification. The overall level of F-actin was compared between amoeba loaded on non-coated and on Fibronectin-coated glass (see Fig 4A). Cell surfaces were detected with HK means plugin of Icy. The sum of fluorescence intensity (Arbitrary Units) per cell area (μm^2^) were Box plotted. F-actin labelling intensity on two substrates were compared by a student t-test: ***: 0.001.
(TIFF)

S1 Video Video of two representative runs within a laminar flow chamber (one on non-coated glass and one on fibronectin). (MOV)

S2 Video Video showing the trajectory of trophozoites migrating on fibronectin (15 frame/seconds). (MOV)

S3 Video Video showing the angle of migration of one trophozoite migrating on non-coated glass and another one on Fibronectin (15 frame/seconds). (MOV)

S4 Video Video showing temporal analysis with Bioflow of the gradient pressure, intracellular flux speed and forces in *E*. *histolytica* migrating on non-coated glass. (MOV)

S5 Video Video showing temporal analysis with Bioflow of the gradient pressure, intracellular flux speed and forces in *E*. *histolytica* migrating on fibronectin. (MOV)
