# Supplementary figures and images for: Fibronectin induces a transition from amoeboid to a fan morphology and modifies migration in Entamoeba histolytica
Source: PLoS Pathog. 2024 Jul 25;20(7):e1012392. doi: 10.1371/journal.ppat.1012392 (PMC11302856; doi:10.1371/journal.ppat.1012392)

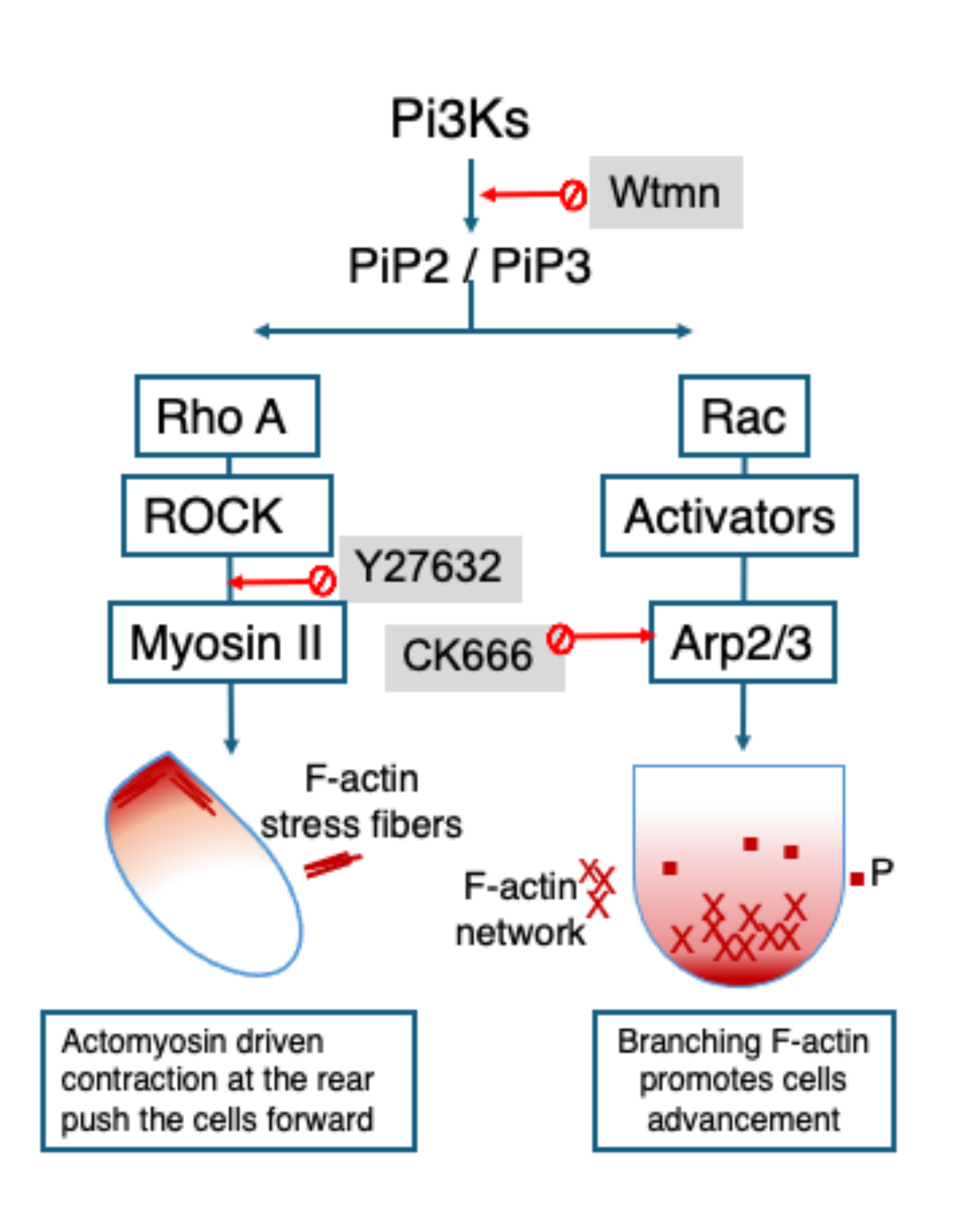

Supplement: S1 Fig — Phosphoinositide 3-kinases (PI3Ks) phosphorylate the membrane lipid phosphatidylinositol-4,5-bisphosphate [PI(4,5)P2] to generate the lipid second messenger phosphatidylinositol-3,4,5-trisphosphate (PIP3) that controls the actin cytoskeleton mainly through activating the guanine exchange factors of small GTPases including Rac1 and RhoA, which govern cell adhesion dynamics and motility. The two most thoroughly characterized types of cell migration are: (i) the amoeboid mode driven by the contractile actomyosin cortex of the cell (left side of the figure). Following RhoA activation of ROCK activities myosin light chain became phosphorylated and the actomyosin functions regulates F-actin stress fibre dynamics and focal adhesions. (ii) the mesenchymal adhesion-dependent migration mode (right side of the figure). Depending on the activity of Rac1 on various intermediate effectors, the Arp2/3 complex nucleates and branches actin filaments forming networks of fine sheet-like lamellipodial protrusions at the cell leading edge and adhesive structures as podosomes (P). Wortmannin (Wtmn) inhibit Pi3Ks, Y27632 inhibit ROCK, and CK666 inhibit Arp2/3 complex assembly. See references in the main text. (TIFF) [file ppat.1012392.s001.tiff]

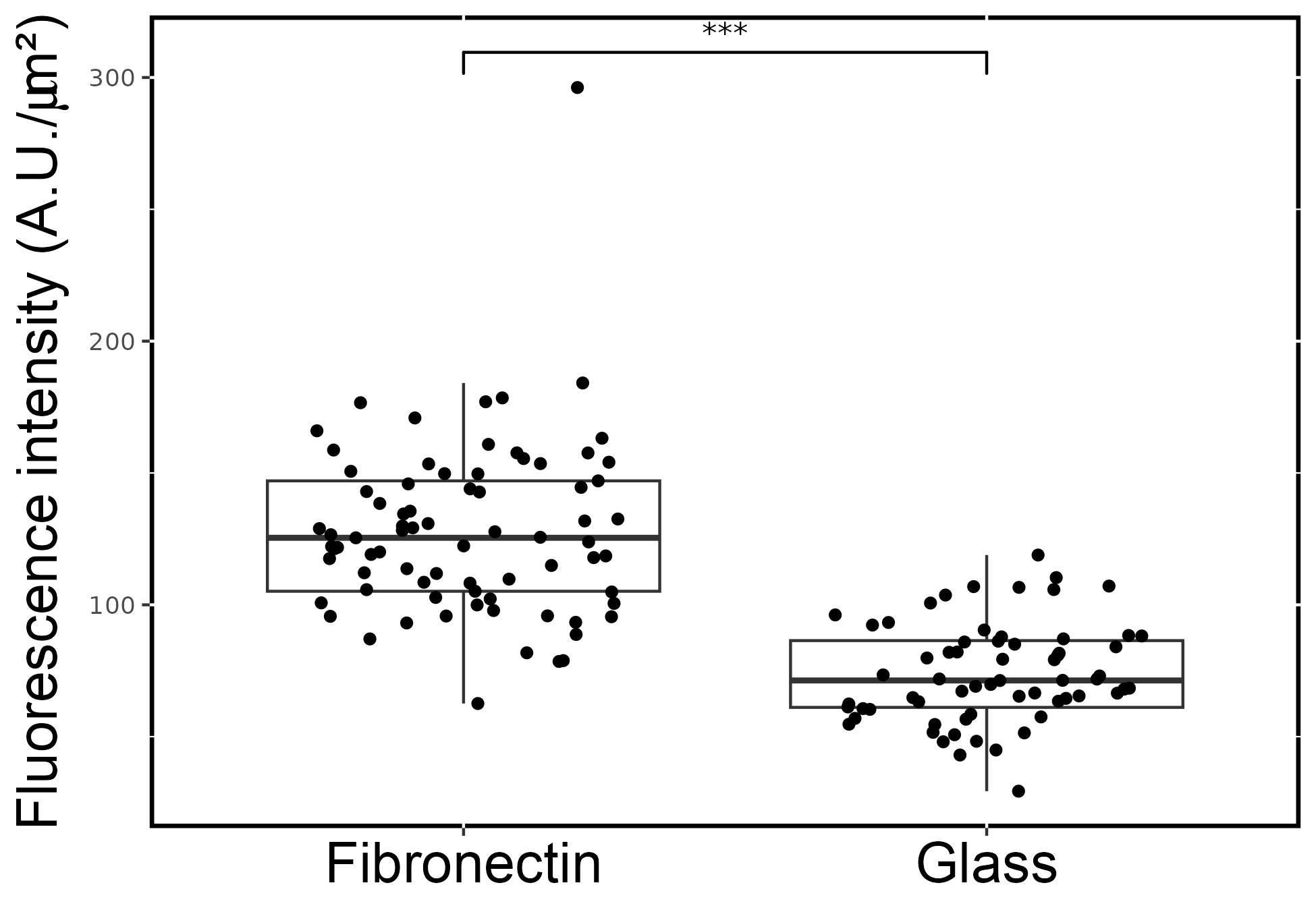

Supplement: S2 Fig — The overall level of F-actin was compared between amoeba loaded on non-coated and on Fibronectin-coated glass (see Fig 4A). Cell surfaces were detected with HK means plugin of Icy. The sum of fluorescence intensity (Arbitrary Units) per cell area (μm2) were Box plotted. F-actin labelling intensity on two substrates were compared by a student t-test: ***: 0.001. (TIFF) [file ppat.1012392.s002.tiff]
